# Chronic recurrent multifocal osteomyelitis beginning with a solitary lesion of the ilium

**DOI:** 10.1186/s12891-017-1611-4

**Published:** 2017-06-06

**Authors:** Ping Zhang, Xiao Ying Jia, Yun Zhang, John Morelli, Ze Kun Zhang

**Affiliations:** 1 Department of Radiology, The third Hospital of Hebei Medical University, Hebei Province Biomechanical Key Laborary of Orthopedics, No.139, Ziqiang Road, Shijiazhuang City, Hebei Province 050051 China; 2 Department of Emergency, The Hebei general Hospital, Shijiazhuang, Hebei 050051 China; 3 Department of Outpatient, Chinese people’s Armed Police Force 8640 hospital, Dingzhou, Hebei China; 4 Department of Radiology, St. Johns Medical Center, Tulsa, OK USA

**Keywords:** Chronic recurrent multifocal osteomyelitis, Ilium, Periosteal proliferation

## Abstract

**Background:**

Chronic recurrent multifocal osteomyelitis (CRMO) is an idiopathic inflammatory disease. The initial lesions are typically found in the metaphyses, generally without periosteal reaction.

**Case presentation:**

We present a case of a 14-year-old female teenager with relapsing and remitting right iliac pain. There was no evidence of infectious organisms, neoplastic processes, or hematologic malignancy based on laboratory tests. Initial computed tomography (CT) and magnetic resonance imaging (MRI) demonstrated atypical periosteal proliferation in the right ilium. Histopathology demonstrated only non-specific chronic inflammation compatible with CRMO. Two years later, this patient developed left humeral pain. MRI and CT images revealed thickening and marrow edema involving the humeral cortex.

**Conclusions:**

This case highlights that CRMO can begin as a unifocal lesion and also possibly within the ilium, despite usually being multifocal and involving the long bone metaphysis.

## Background

Chronic recurrent multifocal osteomyelitis (CRMO) is an idiopathic inflammatory disease, also known as chronic nonbacterial osteomyelitis. It was first described in 1972 by Giedion et al., in 4 cases of patients suffering from “symmetrical” bone pain [1]. However, the clinical presentation of CRMO has greater diversity. It is best characterized as a skeletal disorder resulting in episodic bone pain of unknown cause. The prevalence of CRMO is estimated as less than 1 in 1,000,000. It mainly affects children and adolescents, especially females (up to 85%). The lesions are mainly located in the metaphyses and epiphyses of the long bones [2–6]. Some patients might have extra-articular manifestations such as palmoplantar pustulosis, psoriasis, Crohn’s disease and other entities [7, 8]. Radiography is the primary examination in pediatric patients presenting with a limp or pain possibly originating in bone. The initial lesions of CRMO are usually found in the metaphyses, near the growth plates, generally without periosteal reaction. If the lesions have breached the cortices with or without periosteal reaction, they may be misdiagnosed as a tumor, especially the unifocal lesions [9–11].

Here, we describe the case of a 14-year-old girl who initially presented with episodic osseous right ilium bone pain, eventually diagnosed with CRMO after extensive evaluation. The diagnosis in this case was difficult to make because of the unusual initial site and atypical presentation of periosteal proliferation and soft tissue swelling. Knowledge of this unusual case may help skeletal radiologists to consider CRMO as one of the differential diagnoses in patients with similar presentations.

## Case presentation

In July 2013, a 14-year-old girl without history of trauma or medication use presented with a one-year-history of right hip pain resulting in limitations of daily activities. The pain was relapsing and remitting in nature, without any associated numbness, weakness, or bowel dysfunction. There was no history of fever, no other musculoskeletal complaints, and no family history of skeletal problems.

On physical examination, the patient experienced obvious pain in the right iliac wing on palpation and had a positive Patrick’s test (A test performed by having the tested leg flexed and the thigh abducted and externally rotated. If pain is elicited, it is suggestive of a hip joint or the sacroiliac joint disorder). The right hip had a range of motion of approximately 90 degrees of flexion, 10 degrees of abduction, 20 degrees of internal rotation and 30 degrees of external rotation. The skin temperature was normal. Laboratory results revealed a normal white blood cell count, mildly elevated C-reactive protein (CRP) of 16.57 mg/L (normal value, ≤10 mg/L), and normal erythrocyte sedimentation rate (ESR) of 12.65 mm/h (normal value, 0-20 mm/h).

### Imaging findings

**Fig. 1:**
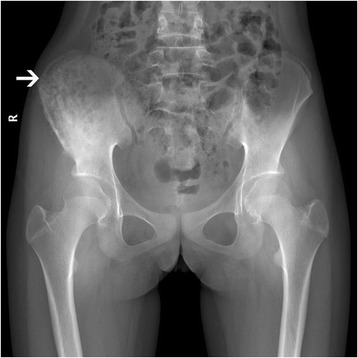
Anteroposterior radiograph of pelvis shows a predominantly lytic process with sclerosis and periosteal proliferation in the right iliac wing (*white arrow*)

**Fig. 2:**
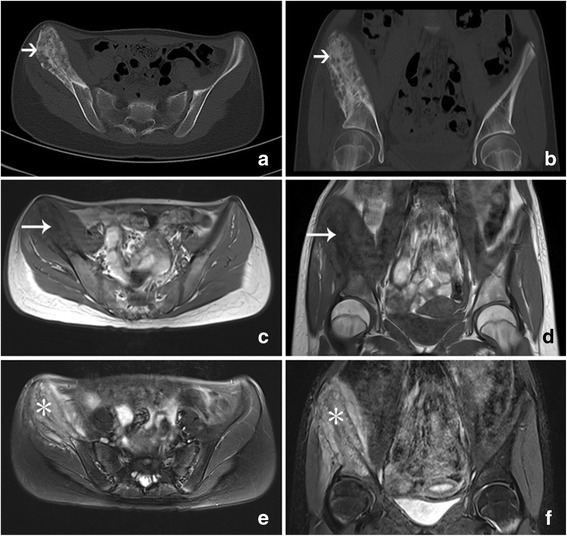
Axial (**a**) and reformatted coronal (**b**) CT images obtained in bone windows illustrate an osteolytic, expanded lesion with mild sclerosis and periosteal proliferation (*short arrow*). MRI of this lesion presented hypo-signal intensity (*long arrow*) on T1-weighted images (**c, d**) and scattered hyper-signal intensity (*star*) on fat suppressed T2-weighted images (**e, f**), with soft tissue edema

**Fig. 3:**
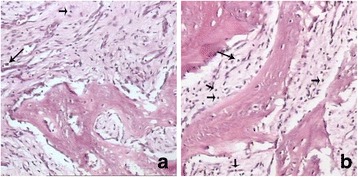
Microscopic examination of the right ilium biopsy (HE 10 × 10) shows a non-specific fibro-inflammatory infiltrate composed of scattered chronic inflammatory cells including lymphocyte neutrophils (*short arrow* **a**, **b**) and plasma cells (*long arrow* **a**, **b**)

Initial radiography demonstrated a mixed lytic and sclerotic lesion of the right ilium with extensive periosteal reaction, suggesting an aggressive process such as a neoplasm (Fig. 1). The MRI revealed that the right ilium was expanded with diffuse bone marrow edema and adjacent soft tissue edema within the iliacus and gluteus minimus muscles (Fig. 2). Findings were concerning for an infiltrative or malignant process, such as lymphoma, Ewing’s sarcoma or osteomyelitis. A CT scan demonstrated a markedly atypical periosteal proliferation involving the right ilium with a multifocal lytic lesion (Fig. 2). An open biopsy was subsequently performed followed by surgical bone curettage of right ilium. Histopathology showed a nonspecific fibro-inflammatory infiltrate composed of scattered chronic inflammatory cells including lymphocyte and plasma cells (Fig. 3). There was no evidence of neoplasm. Antibiotic therapy was started with intravenous ceftriaxone and clindamycin hydrochloride. After one week of antibiotic therapy, the patent’s clinical signs and symptoms resolved.

### Subsequent imaging, pathology, and laboratory findings

**Fig. 4:**
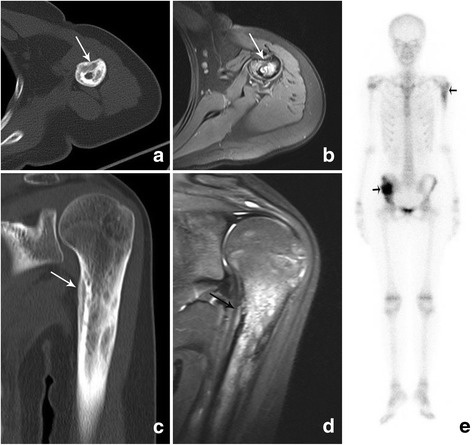
CT images of the left humerus (**a, c**) obtained with bone window and fat-suppressed T2-weighted MR images (**b, d**) illustrate an osteolytic lesion with periosteal proliferation (*white arrow*) similar to the initial lesion. Techenetium-99 m methylene diphosphonate bone scintigraphy (**e**), anterior view of the whole body presents increased radiotracer activity within the right ilium and left humerus (*black arrow*)

In July 2015, the patient was readmitted due to left upper arm pain and a mild fever. On physical examination, there was localized tenderness to the left arm. Full blood count was again normal. The CRP was 58.65 mg/L (normal value, ≤10 mg/L), and the ESR was 110 mm/h (normal value, 0-20 mm/h). Blood cultures were negative. The upper extremity MRI revealed a left humeral lesion with diffuse low T1 and intermediate to hyper intense T2 signal. There was no evidence of abscess formation in the surrounding soft tissue structures. CT revealed obvious cortical thickening of the left humerus (Fig. 4). A whole-body Technetium-99 m hydroxymethyl disphosphonate (Tc-99mHDP) bone scan was performed to investigate the possibility of multifocal lesions. It showed mild increased activity in the left humerus and obviously increased activity in the right ilium (Fig. 4). The bone biopsy of the left humerus revealed a non-specific fibro-inflammatory infiltrate and normal hematopoietic marrow without organism growth or evidence of neoplasm. Acid fast and KoH stains were negative. The flow cytometric analysis showed no evidence of a hematologic malignancy.

### Clinical diagnosis

The biopsies of the lesions were notably negative for malignancy or infection. Based on the progressive nature of the patient’s clinical symptoms and imaging findings, she was diagnosed with CRMO. The treatment of CRMO generally involves anti-inflammatory agents targeting symptomatic relief, particularly non-steroidal anti-inflammatory medication (i.e. ibuprofen). The patient experienced relief with anti-inflammatory therapy and with local treatment of the right iliac lesion, leading support to the diagnosis.

## Discussion and Conclusions

CRMO is a rare inflammatory bone disease, accounting for 2% to 5% of all cases of osteomyelitis [12]. Currently the etiology of CRMO is unknown. Several studies are in progress to investigate its pathogenesis. The generally supported hypothesis suggests a noninfectious inflammatory process as a possible etiology due to the fact that pathogens cannot be found in blood cultures and antibiotics cannot relieve symptoms or alter the disease course [13, 14]. Autoimmune causes have also been hypothesized as a possible etiology. According to reports, CRMO is associated with peripheral arthritis [15], inflammatory bowel disease [2], and sacroiliitis [8]. It might also represent a special type of juvenile seronegative spondyloarthropathy, as it exhibits some features of SAPHO (synovitis, acne, pustulosis, hyperostosis, and osteitis) syndrome [14].

CRMO is primarily a diagnosis of exclusion based on the clinical symptoms, laboratory tests, imaging and pathological findings. In general, the occurrence of multifocal bone pain is suggestive of CRMO, however investigations must be performed to rule out other major differential considerations, such as infectious osteomyelitis and bone tumors [9]. The lesions are typically located in the metaphyses of tubular bones, followed by the clavicle and spine [6, 16, 17]. It is unusual for CRMO to begin in a flat pelvic bone such as the ilium; although an initial presentation in the vertebra has been previously reported in children and adults [18, 19]. It is reported that a CRMO patient with an initial unifocal lesion has a more significant diagnostic delay than a patient with multifocal lesions [4]. The unusual location of the initial solitary lesion (right ilium) in this case added to difficulties in the diagnosis of CRMO. A prolonged and fluctuating course with recurrent episodes of pain is a feature of CRMO. The activity of CRMO may persist for years or even decades. In this case, the left humeral lesion, also contributing to the diagnostic delay.

In general, the initial imaging manifestation of CRMO is lytic destruction in the metaphyses of tubular bones, demarcated by a sclerotic rim. Bone destruction without periosteal reaction or sequestration formation is typical [20–24]. The initial presentation in this case with significant periosteal proliferation made it difficult to rule out a malignant tumor, especially Ewing’s sarcoma and Langerhans’ cell histiocytosis. In CRMO involving tubular bones, abscess formation and soft tissue inflammatory changes are generally absent, whereas in this case there was mild soft tissue inflammation, making it difficult to rule out infectious osteomyelitis. MRI is sensitive to the bone marrow edema and soft tissue inflammatory changes seen in CRMO, but these findings are nonspecific.

Pathologic investigation plays a major role in ruling out other diagnoses, especially when oncologic bone lesions and osteomyelitis cannot be excluded by imaging. Histological investigations show inflammatory changes with granulocytic, lymphocyte, and plasma cell infiltration [25, 26]. It is impossible to cultivate any infectious organism from CRMO, so antibiotic treatment is useless. Reduction of bone pain with anti-inflammatory therapy does not correspond with radiological remission [27]. It is reported that bisphosphonate therapy has a positive effect on CRMO [28, 29]. Unfortunately, this patient did not receive bisphosphonate therapy.

### Conclusions

CRMO has no specific clinical, imaging, or pathological findings. The diagnosis depends on a multidisciplinary effort. It is therefore important for skeletal radiologists to be familiar with the variable presentations of CRMO. This case demonstrates that CRMO can present as a solitary lesion of the ilium with significant periosteal proliferation and soft tissue changes. Knowledge of this unusual case may help skeletal radiologists to consider CRMO in their differential diagnosis.

## Abbreviations

CRMO: Chronic recurrent multifocal osteomyelitis
CT: Computed tomography
MRI: Magnetic resonance imaging
CRP: C-reactive protein
ESR: Erythrocyte sedimentation rate
Tc-99mHDP: Technetium-99 m hydroxymethyl disphosphonates
SAPHO: Synovitis, acne, pustulosis, hyperostosis, and osteitis
